# Accuracy of preimplantation genetic screening (PGS) is compromised by degree of mosaicism of human embryos

**DOI:** 10.1186/s12958-016-0193-6

**Published:** 2016-09-05

**Authors:** Norbert Gleicher, Andrea Vidali, Jeffrey Braverman, Vitaly A. Kushnir, David H. Barad, Cynthia Hudson, Yang-Guan Wu, Qi Wang, Lin Zhang, David F. Albertini, the International PGS Consortium Study Group, Norbert Gleicher, Andrea Vidali, Jeffrey Braverman, Vitaly A. Kushnir, David H. Barad

**Affiliations:** 1 The Center for Human Reproduction, 21 East 69th Street, New York, NY 10021 USA; 2 The Foundation for Reproductive Medicine, New York, NY USA; 3 The Brivanlou Laboratory for Stem Cell Biology and Molecular Embryology, Rockefeller University, New York, NY USA; 4 Fertility Specialist in New York, New York, NY USA; 5 Braverman IVF & Reproductive Immunology, New York, NY USA; 6 Department of Obstetrics and Gynecology, Wake Forest University, Winston Salem, NC USA; 7 Department of Obstetrics and Gynecology, Albert Einstein College of Medicine, Bronx, NY USA; 8 Department of Molecular and Integrative Physiology, University of Kansas School of Medicine, Wichita, KS USA

**Keywords:** Preimplantation genetic screening (PGS), In vitro fertilization (IVF), Embryos, Embryo mosaicism, Trophectoderm biopsy, Blastocyst

## Abstract

**Background:**

To preclude transfer of aneuploid embryos, current preimplantation genetic screening (PGS) usually involves one trophectoderm biopsy at blastocyst stage, assumed to represent embryo ploidy. Whether one such biopsy can correctly assess embryo ploidy has recently, however, been questioned.

**Methods:**

This descriptive study investigated accuracy of PGS in two ways. Part I: Two infertile couples donated 11 embryos, previously diagnosed as aneuploid and, therefore, destined to be discarded. They were dissected into 37 anonymized specimens, and sent to another national laboratory for repeat analyses to assess (i) inter-laboratory congruity and (ii) intra-embryo congruity of multiple embryo biopsies in a single laboratory. Part II: Reports on human IVF cycle outcomes after transfer of allegedly aneuploid embryos into 8 infertile patients.

**Results:**

Only 2/11 (18.2 %) embryos were identically assessed at two PGS laboratories; 4/11 (36.4 %), on repeat analysis were chromosomally normal, 2 mosaic normal/abnormal, and 5/11 (45.5 %) completely differed in reported aneuploidies. In intra-embryo analyses, 5/10 (50 %) differed between biopsy sites. Eight transfers of previously reported aneuploid embryos resulted in 5 chromosomally normal pregnancies, 4 delivered and 1 ongoing. Three patients did not conceive, though 1 among them experienced a chemical pregnancy.

**Conclusions:**

Though populations of both study parts are too small to draw statistically adequately powered conclusions on specific degrees of inaccuracy of PGS, here presented results do raise concerns especially about false-positive diagnoses. While inter-laboratory variations may at least partially be explained by different diagnostic platforms utilized, they cannot explain observed intra-embryo variations, suggesting more frequent trophectoderm mosiaicsm than previously reported. Together with recentl published mouse studies of lineages-specific degrees of survival of aneuploid cells in early stage embryos, these results call into question the biological basis of PGS, based on the assumption that a single trophectoderm biopsy can reliably determine embryo ploidy.

## Background

Human embryos are frequently aneuploid. The prevalence further increases with advancing female age and with low functional ovarian reserve (LFOR) [1]. Avoiding transfers of aneuploid embryos in association with in vitro fertilization (IVF) has been proposed since the early 1990s [2] under the assumption it would lead to better pregnancy rates and fewer miscarriages [3]. This effort was given the acronym preimplantation genetic screening (PGS) but over almost two decades has failed to demonstrate promised improvements of in vitro fertilization (IVF) outcomes.

Initial criticisms of the procedure [4–7] were attributed to technical shortcomings of initial cleavage-stage embryo biopsies and inadequate techniques of chromosomal analyses [8]. Once embryo biopsy moved from cleavage- to blastocyst-stage (trophectoderm), and selected chromosome investigation to full chromosomal complement analyses with highly accurate newly developed diagnostic platforms, the widely held assumption was that PGS, finally, would show its clinical effectiveness. When this did not happen, the ability of different PGS platforms to accurately determine embryo ploidy was questioned [9].

Observing in clinical practice statistically improbable high aneuploidy rates, especially in some younger women, The Center for Human Reproduction (CHR) decided in 2014 to offer women with only aneuploid embryos embryo transfers with allegedly aneuploid embryos in selected cases [10], an effort quickly joined by other fertility centers, leading to the establishment of the International PGS Consortium, dedicated to investigations of effectiveness of PGS in association with IVF.

The Consortium since reported 3 chromosomally normal live births from 5 such transfers [11]. Concomitantly, an Israeli member of the International PGS Consortium completed a PGS study of embryos with single gene diseases, reporting significant discrepancies between multiple trophectoderm and inner cell mass biopsies (Prof. Raoul Orvieto, personal communication, New York, October 2015). This study has since been published [12]. Shortly thereafter, Italian colleagues reported on 18 attempts of transfer of “mosaic” (i.e., aneuploidy) embryos in women who had produced no euploid embryos in IVF cycles, establishing 6 chromosomally normal live births (33.3 %) [13].

This manuscript now raises additional doubts about the ability of PGS to accurately determine embryos ploidy by reporting significant inter-laboratory and intra-embryo discrepancies for PGS results as well as two additional normal pregnancies established from transfer of embryos previously designated as aneuploid.

## Methods

Here reported study received Institutioanl Review Board (IRB) approval ER 10232015:01 at The Center for Human Reproduction.

### PGS laboratories and PGS platforms

The CHR received 11 donated embryos for Part 1 of here reported manuscript. These embryos had previously at two national referral laboratories of international repute (both laboratories have extensively contributed to the PGS literature) been reported as aneuploid: Reprogenetics (Livingston, N.J., now a division of Cooper Surgical, Trumbull, CT) served one couple utilizing array CGH [14], and the not-for-profit Foundation for Embryonic Competence (FEC), utilizing an in-house developed test, called Select Comprehensive Chromosome Screening (CCS) based on real-time polymerase chain reaction (qPCR) technology, developed by Reproductive Medicine Associates of New Jersey (Basking Ridge, N.J.) [15] served the other couple.

### Specimen preparation and coding

All donated embryos investigated in this study were donated to The Center for Human Reproduction (CHR). Most were donations of the center’s own patients but some were donated by a couple that had their embryos stored at another Consortium member’s laboratory. CHR received these embryos cryopreserved and with full documentation of PGS results from the original IVF center. They were maintained in standard liquid nitrogen tanks until dissection of all 11 embryos into 37 individual specimens (1–5 per embryo, depending on technical feasibility) by one of the authors (C.H.) Fragmentation of embryos was achieved utilizing standard laser-assisted dissection instrumentation, as is routinely utilized for trophectoderm biopsies during PGS.

In mimickery routine, the 37 specimens were randomly coded as 4 patients (in Table 1, A1-A8, B1-B8, C1-C10 and D1-D11) and sent to a third national PGS laboratory (IVIGEN, Miami, FL). This laboratory used for 24-chromosome assessment comparative genomic hybridization (aCGH, BlueGnome, 24sure BAC-based arrays), in more detail described at: http://www.cytochip.com.

**Table 1:** Comparison of embryo ploidy between two PGS 2.0 assessments

Pat.#	Emb#	Biopsy #	Original PGS analysis (all embryos reported as abnormal)	Repeat PGS analysis^a^ (multiple biopsies)
1	A1	1	45, XY, -18^b^	Normal 46, XX
2	A2	1	Complex aneuploid^b^	XY, +10, -18q
	A3	2		XY, +11, +16, -21
	A4	3		XX, -3q
3	A5	1	46, XY, +3, -11, +15, -14^b^	XX, -2
	A6	2		Normal 46XX
	A7	3		45, XY, -18
	A8	4		Normal 46,XX
4	B1	1	46,XY, +3, -11^b^	45, XY, -14
	B2	2		45, XY, -14
	B3	3		45, XY, -14
	B4	4		45, XY, -14
5	B5	1	47,XY, +19^b^	47, XY, +3
	B6	2		47, XY, +3
	B7	3		47, XY, +3
	B8	4		Normal 46, XY
6	C1	1	45, XX, -1^b^	Normal 46, XX
	C2	2		Normal 46, XX
	C3	3		Normal 46, XX
7	C4	1	47, XY, +19^b^	Normal 46, XY
	C5	2		Normal 46, XY
	C6	3		Normal 46, XY
8	C7	1	47, XY, +19^c^	Normal 46, XY
	C8	2		Normal 46, XY
	C9	3		Normal 46, XY
	C10	4		Normal 46, XY
9	D1	1	Complex aneuploid^c^	Normal 46, XY
	D2	2		47, +18
10	D3	1	Complex aneuploid^c^	47, XY, +8q, -15, +16
	D4	2		46, XY, -15, +16
	D5	3		46, XY, -15, +16
	D6	4		46, XY, -15, +16
	D7	5		46, XY, -15, +16
11	D8	1	46, XX, +14, -15^c^	46, XX, +14, -15
	D9	2		46, XX, +14, -15
	D10	3		46, XX, +14, -15
	D11	4		46, XX, +14, -15

*Pat#* patient number, *Emb#* embryo number; The diagnostic platforms utilized by the various PGS laboratories are described under Methods: ^a^aCGH, ^b^qPCR and ^c^aray CGH

The laboratory was aware that submitted specimens involved a research project, but was uninformed about details and purpose of the project. Specimen codes were broken upon receipt of results, at which time inter-laboratory and intra-embryo/laboratory discrepancies were determined.

### Embryo transfers

Transfers of supposedly chromosomally abnormal) embryos were performed at three independent fertility centers in New York City, CHR, Fertility Specialists in New York and Braverman IVF & Reproductive Immunology. CHR developed and published in 2014 a policy, since also adopted by the other IVF centers [10], which with appropriate and detailed informed consent allowed transfer of selected supposedly aneuploid embryos if no or only inadequate numbers of euploid embryos were available for transfer.

### Statistical considerations

Because of small numbers of involved patients and embryo specimens, statistical comparisons and determinations of assciations were not possible. Since embryos diagnosed to be aneuploid usually, however, are ethically discarded, reported live births after transfer of such embryos categorically reflect otherwise unachievable outcomes.

## Results

Table 1 summarizes the chromosomal abnormalities of 11 embryos in their original testing round, and in their subsequent repeat evaluation, dissected into 1–5 fragments.

### Outcome comparison between PGS laboratories

Only 2/11 (18.2 %) of embryos demonstrated congruent results between both laboratory evaluations: embryo A2-A4 and D8-D11. Most remarkably, 4/11 (36.4 %) of embryos, originally reported as abnormal, on repeat assessment were found to be normal 46, XX or 46, XY embryos (A1, C1-C3, C4-C6 and C7-C10). An additional 2 embryos, at least in some biopsies demonstrated normal karyotypes (A6 and A8, B8). Combined, 6/11 (54.6 %) embryos upon retesting were either definitely normal or mosaic with potential to be normal, thus offering potential pregnancy chances if transferred. Even where both laboratories agreed that embryos were chromosomally abnormal, there was complete congruence between both laboratories in only 1/11 (9.1 %) embryos (D8-D11). Remarkably, even sex chromosome identifications were apparently inaccurate since they varied between laboratories.

### Intra-embryo variation based on multiple biopsies

Among 10 embryos where more than one biopsy was submitted for PGS in the second testing round, 5 (50 %) offered congruent results on 2–4 biopsies (B1-B4, C1-C3, C4-C6, C7-C10 and D8-D11). All other embryos, even in the same PGS laboratory, demonstrated varying outcomes at different biopsy areas. Intra-embryo differences also applied to sex chromosomes. These outcome data produced in a single laboratory, nevertheless, suggest a higher degree of repoducability than outcome comparisons between different laboratories demonstrated, using different diagnostic platforms.

The small number of so evaluated embryos does not permit ultimate conclusions about what likely mosaicism rates in human embryos may be. Here presented data, do, however, suggest that they are significantly higher than the 4.8 % rate recently detected by Greco et al. [13], and more in line with the 2/5 (40 %) range recently reported by Tiegs et al. [16]. It is also important to consider that these rates may be maternal age-dependent.

### Outcome of transfer of (by PGS.2.0) aneuploid embryos

So far 11 women have qualified for transfer of allegedly aneuploid embryos. Among those, 8 decided to undergo embryo transfers, and 5 conceived. All 5 have since delivered healthy offspring. All pregnancies were confirmed to be chromosomally normal with no evidence of mosaicism. The other three patients did not conceive, though one experienced a chemical pregnancy and, therefore, apparently had a short-lived implantation. Pregnancies are presented in more detail in Table 2.

**Table 2:** Characteristics of aneuploid embryos transferred that led to implantation

Patient	n Embryos transferred	Embryos transferred	Outcome
1	1	43, XY, -13, -15, -18	Normal birth, 46, XY
2	1	45, XY, -21	Normal birth, 46, XY
3	2^a^	45, XY, -21 46, XX	Normal birth, 46, XY
4	2^b^	Partial 47, XX,17p11.2-pter 45, XY, -22	Normal ongoing 46, XX
5	2^c^	47, XY, +22 Partial 45, XY,-1plar-p36, 12	Normal ongoing 46, XY
6	1^d^	45, XY, -21	Chemical pregnancy

^a^This patient, who had undergone PGS for sex selection (desired sex male), had a 45, XY, -21 and a normal 46, XX female transferred. Since she delivered a healthy male, the pregnancy had to be the result of the 45, XY, -21 embryo

^b^Two embryos were transferred; normal 46, XX per CVS. Pregnancy, therefore, had to arise from partial trisomic embryo transferred. Currently 20 weeks

^c^Two embryos transferred; normal 46, XY per amniocentesis. Embryo leading to pregnancy unknown; Currently 19 weeks

^d^Chemical pregnancy indicates implantation but not considered a clinical pregnancy; Ploidy unknown

Here observed rate of 5/8 (62.5 %) of embryo transfers leading to live birth or normally progressing euploid pregnancies in a highly unfavorable patient population with minimal embryo yields in IVF cycles, is unexpectedly high. Considering the small patient numbers, percentages may, therefore, represent statistical aberrations, Greco et al. recently also reported a high live birth rate of 33 % in a similarly unfavorable patient population after embryo transfers with known mosaic embryos. Lke we here, they reported no miscarraiges [13]. Eventhough the combined data from both study groups are still limited, they, at minimum, contradict that the transfer of mosaic embryos either impedes implantation or increases clinical miscarriage risks, as was recently suggested [17].

## Discussion

### Principal findings

Considering growing clinical discomfort with the increasing utilization of PGS in IVF and recently arising questions about accuracy of the procedure, this study attempted to assess the clinical reproducibility of PGS results. To bring some clarity to the subject, we dissected 11 embryos previously reported to be aneuploid. To our surprise, a repeat analysis by a third laboratory was congruent in only 2/11 (18.2 %) embryos. Indeed, 4/11 embryos (36.4 %) were on repeat evaluation normal 46, XX and 46, XY, and an additional 2/11 embryos were mosaic, with at least one fragment reported as normal 46, XX or 46, XY.

Though here investigated embryo numbers were small and, therefore, do not allow for statistically valid prevalence assessments, combined, these numbers suggest a potential false-positive PGS rate of as high as almost 55 %. Moreover, intra-embryo discrepancies were observed in 50 % (5/10) of embryos suggesting much higher trophectoderm mosaicism in blastocyst-stage embryos than has previously reported.

### Strength and limitations

Greco et al. recently reported only a 4.8 % mosaicism rate in 3802 blastocyst stage embryos they examined by single trophectoderm biopsy [13]. That this represents a significant underestimate is suggested by an earlier study of Fragouli et al. who reported 32.4 % among 52 investigated blastocyst stage embryos to be mosaic, 30 % uniformly aneuploid and 42.3 % uniformly euploid [17]. This discrepancy in mosaicism rates can, likely, be explained by Greco et al. performing only a single trophectoderm biopsy, while Fragoulis et al., like we in this study, dissected embryos.

A single trophectoderm biopsy usually includes only approximately 5–6 cells, while the whole trophectoderm contains at that stage a few hundred. This consideration alone demonstrates how low the statistical likelihood of detecting mosaicism (i.e., presence of chromosomally normal and abnormal cells) is within a single trophectoderm biopsy. The likelihood of detecting mosaicism will, however increase with increasing numbers of biopsies, both within individual biopsies but also between biopsies at different areas since those may represent euploid and/or aneuploid cell lineages of trophectoderm.

Bolton et al. recently demonstrated in a mouse model that the fate of aneuploid cells in early embryos depends on lineage: aneuploid cells in the fetal lineage (i.e., inner cell mass) are eliminated by apoptosis, whereas those in the placental lineage (i.e, trophectoderm) demonstrate severe proliferative defects. From blastocyst stage on aneuploid cells progressively deplete, and mosaic embryos have full developmental potential as long as they contain sufficient euploid cells [18]. With aneuploid cells preferably seggregating into the trophectoderm, it appears reasonable to assume that mosaicism rates will increase with number of trophectoderm biopsies performed. This was also suggested by the recent study of Orvieto et al. [12].

Under the assumption of a relatively high mosaicism rate in trophectoderm, the biological plausibility of PGS to reliably determine ploidy of embryos with only one trophectoderm biopsy, therefore, has to be questioned. The difference in mosaicism prevalence between Greco’s study on one [13], and Fragouli’s [17] and our study on the other hand, likely, offers a good estimate of how much mosaicism is missed by only a single, randomly chosen trophectoderm biopsy.

As the recent study by Bolton et al. [18] and our study, however, suggest, Fragouli et al., erred when concluding that the significant embryo mosaicism they reported was clinically irrelevant for the accuracy of PGS since only 17 % of mosaic embryos contained also normal cells. They also appear incorrect when concluding that mosaic embryos, therefore, only unlikely would be able to lead to pregnancy and, if they did, pregnancies would, likely, end in miscarriages [17]. As Bolton et al. demonstrated in the mouse [18], and Greco et al. [13] and the PGS Consortium here, mosaic embryos appear to exhibit rather surprising developmental potential.

Combining Greco’s data [13] with here reported cases, so far 26 infertile women worldwide received allegedly aneuploid (and/or mosaic) embryos, resulting in 11 chromosomally normal live births and/or ongoing clinical pregnancies, for a rather remarkable rate of 42.3 % of likely live birth in absence of even a single miscarriage. These unexpectedly high live birth rates in both studies are that more noteworthy since, as demonstrated by small number of available embryos, they involved women with very LFOR. Such patients iusually produce extremely low live birth and very high miscarriage rates [1].

The ultimate pregnancy potential from transfer of mosaic embryos has still to be determined. It, likely, will depend on patient age, a woman’s FOR and the specific aneuploidy/mosaicism pattern an embryo presents with. Studies to obtain this information appear of utmost importance. The International PGS Consortium, therefore, established a PGS Registry for Transfer of Aneuploid/Mosaic Embryos, which can be accessed at www.centerforhumanreprod.com or by e-mailing questions to ykizawa@thechr.com.

## Conclusions

Though differences in diagnostic platforms utilized at different PGS centers may contribute to inter-center variability of observed results, they do not explain the intra-embryo variability of 50 % observed in one PGS center utilizing one platforms for all analyses. Those have to be the result of embryo mosaicism, and such mosaicism can obviously lead to false-positive trophectoderm biopsies. As first reported by Mastenbroek et al. [4], and since confirmed by others [6, 7], poor prognosis patients, who usually produce only small embryo numbers, will be especially harmed by false-positive diagnoses and the discarding of potentially perfectly normal embryos. A moratorium on the utilization of PGS In poor prognosis patients, therefore, appears sensible at this point.

Finally, mosaic aneuploidy and uniparental disomy routinely arise from mitotic as well as meiotic events in human embryos [19]. Even normal human pluripotent stem cell lines apparently exhibit pervasive mosaic aneuploidy [20]. And the opposite is true as well: aneuploid embryos have been reported to have given rise to euploid human embryonic stem cells [21].

Substantial embryonic trophectoderm mosaicism may, therefore, represent a normal feature of early embryo development. Indeed, one could further hypothesize that it may have developmental purposes in facilitating implantation, as aneuploidy has been associated with tumor invasion [22]. This hypothesis, indeed, would explain the high prevalence of here reported mosaicism and the unexpectedly high implantation rates observed by us and Greco et al. [13] in very adversely selected patients. This hypothesis, of course, turns upside down the current rational for PGS because it would suggest potential benefits from embryo selection based on implantation-enhancing trophectoderm mosaicism.

In summary, this study offers further evidence that the increasing utilization of PGS requires careful reassessment. Especially poor prognosis patients appear at significant risk to actually reduce their pregnancy chances. But better and even best prognosis patients may also be negatively affected by PGS, a question we currentlt are exploring further. The primary reason appears to lie in higher than previously appreciated mosaicism of human embryos, which can lead to false positive diagnoses of aneuploidy and the discarding of potentially normal embryos.

Here presented data, however, cannot preclude the possibility that the accuracy of diagnostic platforms employed by laboratories varies in addition. Here presented data, however, are not sufficient to comment on the relative accuracy of various diagnostic platforms, lie aCGH and NGS, currently utilized as part of PGS.

Combined with above quoted recently published studies, here presented data demonstrate with unusal clarity that prudence has to be exercised by practitioners in IVF across all patients when offering PGS under the hypothesis that the procedure improves IVF outcomes.

## Abbreviations

aCGH: Array comparative genomic hybridization
FOR: Functional ovarian reserve
IVF: In vitro fertilization
LFOR: Low functional ovarian reserve
PGS: Preimplantation genetic screening
